# Parathyroid Carcinoma Disseminated Following Endoscopically-Assisted Surgery: A Case Report

**DOI:** 10.70352/scrj.cr.26-0159

**Published:** 2026-05-12

**Authors:** Yoko Omi, Yuki Yamanashi, Tomoyoshi Nakai, Juro Yanagida, Yusaku Yoshida, Naoko Ito, Tomoko Yamamoto, Yoji Nagashima, Kiyomi Horiuchi

**Affiliations:** 1 Departments of Endocrine Surgery, Tokyo Women’s Medical University, Tokyo, Japan; 2 Departments of Surgical Pathology, Tokyo Women’s Medical University, Tokyo, Japan

**Keywords:** parathyroid carcinoma, dissemination, endoscopic surgery, tumor mutational burden high, pembrolizumab

## Abstract

**INTRODUCTION:**

Endoscopically assisted surgery for parathyroid tumors has provided excellent cosmetic results and has been covered by Japan’s national insurance system since 2016. However, careful handling is essential because parathyroid tumor cells can easily disseminate.

**CASE PRESENTATION:**

We herein report the case of a 34-year-old woman who developed widespread dissemination of parathyroid carcinoma after endoscopic resection. She initially underwent endoscopically assisted left thyroid lobectomy for an intrathyroidal parathyroid tumor, which was later diagnosed as a carcinoma. Four years later, multiple cervical recurrences and pulmonary metastases were observed. Extensive cervical tumor resection was performed, including *en bloc* removal of the invaded structures, followed by resection of lung metastases. Despite temporary improvement, the disease progressed. Genomic profiling revealed a *CDC73* mutation and high tumor mutation burden. Pembrolizumab therapy achieved temporary stabilization but was eventually ineffective. The patient died during palliative care.

**CONCLUSIONS:**

This case highlights the risk of tumor cell dissemination in parathyroid surgery when capsular integrity is compromised. While this risk exists regardless of the surgical approach, certain technical factors associated with endoscopically assisted procedures may increase the likelihood of inadvertent capsular injury. Complete *en bloc* resection during the initial operation is essential for parathyroid carcinoma, because incomplete resection leads to poor prognosis. When carcinoma is suspected intraoperatively, surgeons should consider open surgery. Comprehensive genomic profiling may provide therapeutic options such as immune checkpoint inhibitors for recurrent or metastatic cases.

## Abbreviations

i-PTH
: intact parathyroid hormone
MIBI
: ^99m^Tc-methoxy-isobutyl-isonitrile
TMB
: tumor mutational burden

## INTRODUCTION

Endoscopically assisted surgery for parathyroid tumors offers excellent cosmetic outcomes, particularly for patients who are concerned about postoperative neck scars, such as young women. In Japan, endoscopically assisted surgery has been covered by the National Health Insurance System for benign parathyroid tumors since 2016.

Because parathyroid tumor cells are easily disseminated even in benign lesions, it is crucial to avoid damaging the tumor capsule during surgery. We report a case of parathyroid carcinoma that was widely disseminated along the area dissected by endoscopically assisted surgery and highlight the need for caution when applying endoscopic procedures to parathyroid carcinoma.

## CASE PRESENTATION (Fig. 1)

**Fig. 1:**
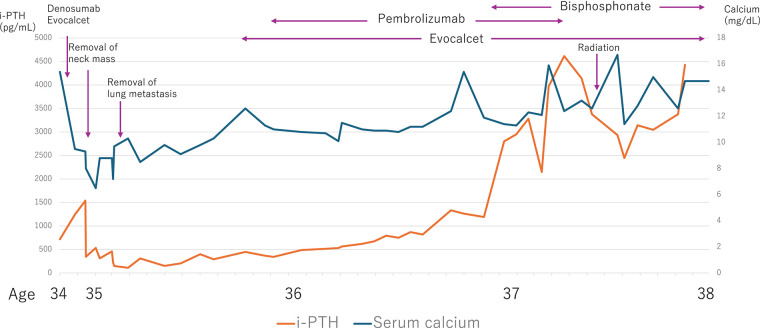
Changes in serum calcium and i-PTH levels and treatment course. i-PTH, intact parathyroid hormone

A 34-year-old woman with a neck mass and hypercalcemia was referred to our hospital. She had undergone endoscopically assisted left thyroid lobectomy for a 4 cm intrathyroidal parathyroid tumor 4 years earlier at another hospital, where the tumor was pathologically diagnosed as parathyroid carcinoma. For this surgery, an anterior thoracic approach via a subclavian incision was employed, and CO_2_ insufflation was used to create a working space. Blood tests were performed every 3 or 4 months postoperatively. Until 3 years and 3 months after surgery, i-PTH remained in the normal range. The patient’s regular hospital visits were interrupted for a year because the surgeon had resigned from the hospital.

Five months prior to her presentation, the patient noticed a palpable mass in her neck and visited the hospital where she had previously undergone surgery (**Fig. 2A**). Laboratory findings revealed a corrected serum calcium level of 15.4 mg/dL and an i-PTH level of 719 pg/mL. As the surgeon who had previously treated her had resigned, she was managed medically by a physician with elcatonin, denosumab, and evocalcet. ^99m^Tc-MIBI scintigraphy demonstrated uptake on the left side of the neck, suggesting recurrence of parathyroid carcinoma (**Fig. 3**). The patient was referred to our hospital for surgical treatment.

**Fig. 2:**
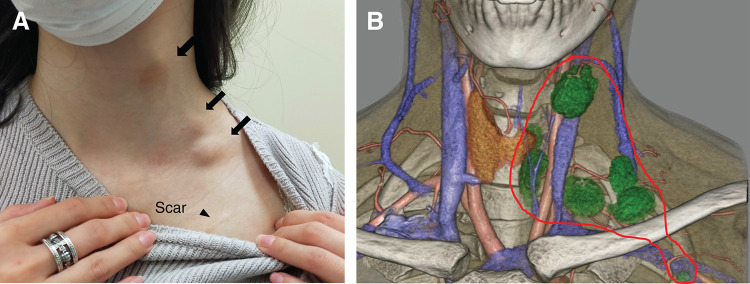
(**A**) Photograph of the patient. Tumors in the left neck (arrows). The arrowhead indicates surgical scar. (**B**) 3D Reconstructed neck CT image. Green indicates the recurrence site. The area outlined in red represents the region that has been resected during the previous surgery.

**Fig. 3:**
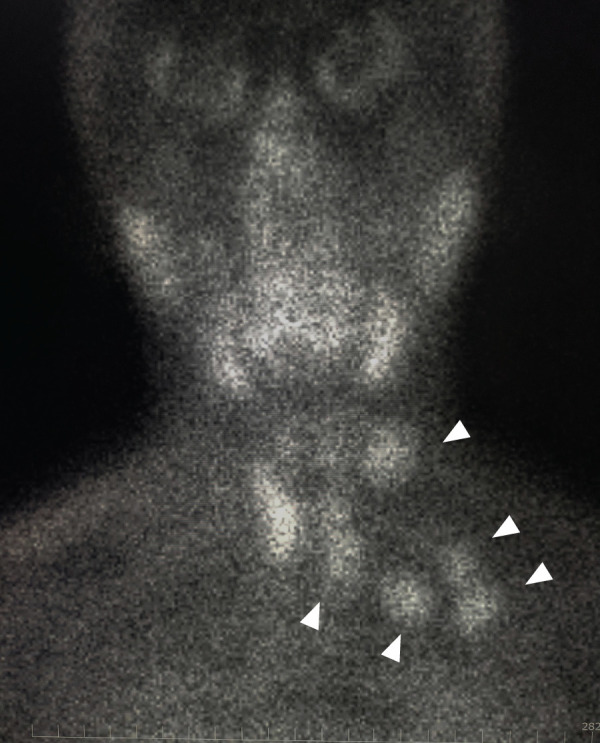
Planar image of ^99m^Tc-MIBI scintigraphy. Uptakes were seen in the recurrences on the left side of the neck (arrowheads). MIBI, ^99m^Tc-methoxy-isobutyl-isonitrile

Contrast-enhanced CT revealed multiple scattered masses, up to 20 mm in size, along the previous dissection area, including the left paratracheal, left submandibular, left upper lateral, supraclavicular, and subcutaneous regions (**Fig. 2B**). Pulmonary metastases were also identified in the right lung (**Fig. 4A**, **4B**).

**Fig. 4:**
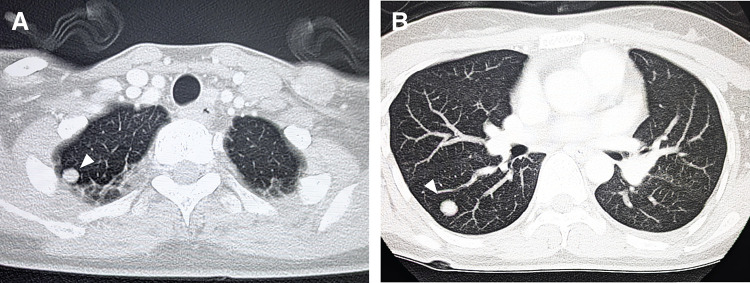
Images of chest CT. (**A**) Pulmonary metastasis in the right upper lobe (arrowhead). (**B**) Pulmonary metastasis in the right lower lobe (arrowhead).

Surgical resection of the cervical tumors was performed as extensively as possible. The left recurrent laryngeal nerve was involved in the paratracheal mass, and the supraclavicular mass invaded the external jugular vein; thus, both structures were resected *en bloc*. The left recurrent laryngeal nerve was reconstructed using end-to-end anastomosis. A histopathological examination revealed solid proliferation of atypical parathyroid cells, confirming recurrent parathyroid carcinoma (**Fig. 5**).

**Fig. 5:**
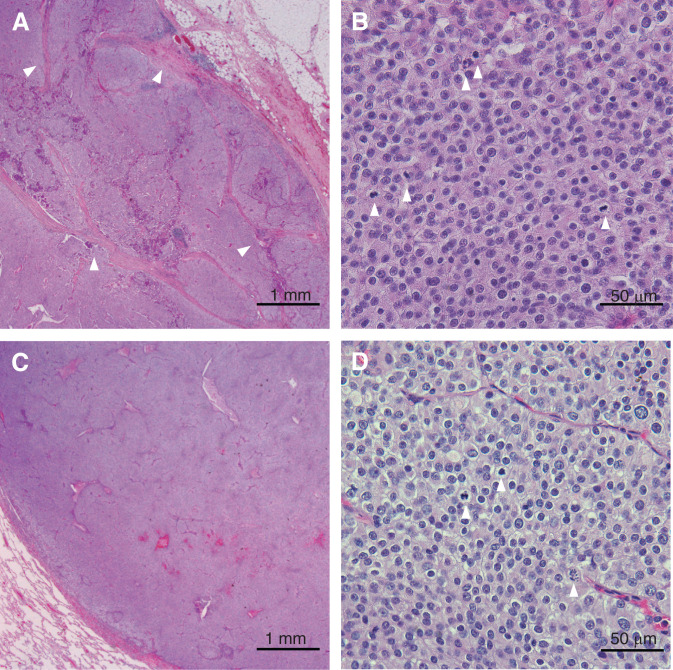
Histological findings. (**A**) Low magnification microscopic image of the recurrence in the neck. Tumor cells show solid growth with fibrous band formation (arrowheads). (**B**) High magnification microscopic image of the recurrence in the neck. The tumor cells do not exhibit significant atypia, but mitotic figures are frequently observed (arrowheads). (**C**) Low magnification microscopic image of the pulmonary metastasis. Like the cervical recurrence, the tumor cells are proliferating in a solid pattern. Fibrosis is not as pronounced in the recurrent neck lesions. (**D**) High magnification microscopic image of the pulmonary metastasis. Atypical tumor cells were observed with frequent mitotic figures (arrowheads).

Postoperatively, her i-PTH level decreased to 346 pg/mL, and she required calcium and vitamin D supplementation to avoid hypocalcemia. Two months later, the patient underwent a right upper lobectomy and enucleation of a metastatic tumor in the right lower lobe. However, her i-PTH and serum calcium levels gradually increased.

Comprehensive genomic profiling (FoundationOne CDx) of the recurrent tumor revealed a *CDC73* gene mutation and a high TMB (13 Muts/Mb). The microsatellite status was stable. As the hypercalcemia became uncontrollable, pembrolizumab therapy (200 mg/3 weeks) was initiated. Hypothyroidism occurred as an immune-related adverse event, requiring 50 mg of levothyroxine. Despite 16 months of treatment, the metastatic lesions in the hilar and supraclavicular lymph nodes and bilateral lungs enlarged, and her i-PTH and serum calcium levels continued to rise. Pembrolizumab was subsequently discontinued owing to its lack of efficacy. External beam radiotherapy (20 Gy) for pulmonary metastases temporarily suppressed the increase in i-PTH levels for approximately 2 months.

Thereafter, her serum calcium level was controlled for approximately 9 months with continuous hydration, bisphosphonate therapy, and evocalcet. Eventually, she became bedridden due to multiple bone fractures and muscle atrophy, and died in hospice care, likely due to renal failure.

## DISCUSSION

It has been 9 years since endoscopically assisted parathyroidectomy was approved for coverage by the National Health Insurance System in Japan. Since then, this procedure has been widely used. Endoscopically assisted parathyroidectomy offers the cosmetic advantage of avoiding a visible neck scar; instead, small incisions are made in the subclavicular area, where they can be easily concealed beneath clothing. It also offers the advantage of a magnified view, which makes precise manual procedures easier to perform. Many reports claim that it is a safe and feasible procedure.^1,2)^ According to a cohort study based on a Japanese claims database, the incidence of complications in endoscopic thyroidectomy and parathyroidectomy was 5.47% for recurrent laryngeal nerve palsy, 5.11% for hypocalcemia, and 1.82% for postoperative hemorrhage.^1)^

Since parathyroid tumor cells can be easily implanted, “parathyromatosis” may occur even in benign parathyroid disease. Although rare, it can lead to recurrent hyperparathyroidism. Parathyromatosis has been reported to be a complication of open parathyroidectomy^3)^ and it can also occur following endoscopic procedures.^4,5)^ Therefore, surgeons must carefully avoid damaging the tumor capsule during the procedure to prevent this complication.

Parathyroid adenomas are typically reddish and soft, without any evidence of invasion into the surrounding tissues, whereas parathyroid carcinomas characteristically appear as firm, whitish lesions, and exhibit adhesions suggestive of invasion into the adjacent structures. Therefore, it is important to assess the color and hardness of the lesions intraoperatively. During endoscopically assisted surgery, tactile sensation is transmitted through endoscopic instruments, potentially causing excessive traction on parathyroid tumors. In addition, it is impossible to directly palpate the hardness by hand or visually confirm the color under direct visualization. Therefore, it may be difficult to recognize hardness and color tone. In comparison to open neck surgery, the neck is more extensively dissected. In this case, CO_2_ gas was used to maintain the working space. Studies using rat models indicate that endoscopic surgery employing CO_2_ gas insufflation has a higher incidence of dissemination than endoscopic surgery without gas use.^6,7)^ If parathyroid tumor cells were spilled under such conditions, they could have been widely disseminated. As a result, the resection in this case was quite extensive. In the present case, although detailed intraoperative findings are unavailable, inadvertent capsular injury during surgical manipulation cannot be excluded and such an injury may have contributed to tumor cell implantation. Because parathyroid carcinoma is a rare disease, no standard treatment other than surgery has been established. Complete resection during initial surgery is crucial to achieving a favorable prognosis.^8,9)^ Therefore, *en bloc* resection is recommended for patients with parathyroid carcinoma. In the present case, left lobectomy was performed together with parathyroidectomy because the preoperative diagnosis was intrathyroidal parathyroid adenoma. However, *en bloc* resection was not intended. Parathyroid carcinoma is often difficult to diagnose preoperatively. If a surgeon recognizes any findings suggestive of parathyroid carcinoma during surgery, such as a firm, whitish tumor with invasion, the surgeon should convert to open neck surgery without hesitation.

Parathyroid carcinoma is a rare disease, and no standardized postoperative follow-up protocol or optimal duration of surveillance has yet been established. In our cohort (excluding the present case), 15 of 38 patients developed persistent or recurrent parathyroid carcinoma during a median follow-up of 31 months, with 6 cases occurring more than 5 years after initial surgery.^10)^ These findings suggest that a 5-year follow-up period may be insufficient. The 5-, 10-, and 20-year relative survival rates were 90%, 86%, and 57%, respectively.^10)^

Although resection should be performed as extensively as possible, even for metastatic or recurrent lesions, there is a limit to surgical management. Fortunately, comprehensive genomic profiling found a high TMB in the recurrent tumor, and pembrolizumab was introduced, which successfully halted disease progression for more than a year. Considering that several studies have reported the efficacy of pembrolizumab in TMB-high parathyroid carcinoma,^11,12)^ comprehensive genomic profiling can provide the benefit of expanding treatment options. Nevertheless, the most critical point in parathyroid tumor surgery is that the initial procedure must be curative. While this principle applies to all surgical approaches, certain technical factors in endoscopic-assisted surgery, such as limited tactile feedback and restricted instrument maneuverability, may increase the risk of inadvertent capsular injury. Therefore, proficient instrument handling techniques are required for endoscopically assisted parathyroidectomy. If such expertise is not available, endoscopic surgery should not be performed. Even with this approach, dissemination of parathyroid tumors remains possible, necessitating strict caution throughout the procedure. If endoscopically assisted surgery carries a risk of capsular injury or is unlikely to achieve curative resection, conversion to open surgery should be considered.

## CONCLUSIONS

Dissemination of parathyroid carcinoma can occur following endoscopic surgery. Surgeons should exercise utmost care to avoid damaging the tumor capsule and aim for complete resection.

## DECLARATIONS

### Availability of data and materials

Data are available from the corresponding author upon request.
